# HCV screening, investigation and management in persons with SUD admitted to Mount Carmel Hospital, Malta

**DOI:** 10.1192/j.eurpsy.2024.836

**Published:** 2024-08-27

**Authors:** M. A. Apap Bologna, K. Sant, A. Camilleri, G. Grech

**Affiliations:** Mount Carmel Hospital, Attard, Malta

## Abstract

**Introduction:**

Individuals who suffer from substance use disorder (SUD) are at increased risk of Hepatitis C (HCV). Mount Carmel Hospital (MCH) is the only public service in-patient mental health care facility on the island of Malta. Individuals with SUD are referred to MCH for support with comorbid mental health conditions.

**Objectives:**

To assess whether current practice meets the UK Clinical Guidelines on Drug Misuse and Dependence (2017) recommendations- yearly screening for HCV, further testing and referral to infectious disease specialists for those who screen positive for HCV. To compare current practice at MCH, Malta with other countries in the European Union and United Kingdom.

**Methods:**

Retrospective analysis of HCV screening, investigation and referral practices as recorded on iSoft Clinical Manager records for SUD-related admissions to MCH under the care of addiction specialists in 2022 (n=120). Admissions data were provided by the data protection office, with permission from the Chairman of the Department of Psychiatry, and de-identified at source to safeguard patient confidentiality.

**Results:**

60% (n=72) of the SUD inpatient population underwent screening for HCV according to guideline recommendations. 37% (n=44) of this cohort has received a positive HCV antibody result. 32 persons had HCV RNA load records, 34% (n=11) of whom had a detectable viral load. 50% (n=17) of those who screened positive for HCV were offered an appointment with an infectious disease specialist within the year, 7 attended. The table below compares HCV status between our group and published data for the UK, Austria and Greece. Despite heterogeneity in study designs and populations (we describe an inpatient cohort with diagnosed SUD, not all of whom inject drugs) comparable proportions have undergone HCV screening in the preceding twelve months and similar proportions have chronic HCV infection.

**Table 1:** 

	% Tested in past year for HCV	% HCV antibody positive	% HCV RNA detectable	% HCV cleared
Malta (MCH 2022)	60	37	34	66
England, Wales, N. Ireland (UAMS 2021)	43	57	26	74
Scotland (NESI 2020)	58	55	81	19
Austria (EMCDDA 2019)	59	85	44	56
Greece (EMCDDA 2019)	/	61	54	46

**Conclusions:**

Most SUD inpatients at MCH undergo HCV screening according to guideline recommendations but current practice falls short of ideal coverage and follow-up care. Current screening practices and chronic HCV infection rates at MCH are comparable to other countries in the EU and UK.

**Disclosure of Interest:**

None Declared

